# Comparative Modelling Techniques: Where are we?

**DOI:** 10.1002/cfg.306

**Published:** 2003-07

**Authors:** Anna Tramontano

**Affiliations:** Department of Biochemical Sciences ‘A. Rossi Fanelli’ University of Rome ‘La Sapienza’ Rome 00185 Italy

## Abstract

The enormous increase in data availability brought about by genomic projects is paralleled by an equally unprecedented increase in the expectations for new medical, pharmacological, environmental and biotechnological discoveries. Whether or not we will be able to meet (at least partially) these expectations will depend on how well we will be able to interpret the data and translate the mono-dimensional information encrypted in genomes into a detailed understanding of its biological meaning at the phenotypic level. The process is far from being trivial, and the obstacles along the road are formidable: even the problem of identifying coding regions in eukaryotic genomes is not completely solved. Far more complex is identification of the function of the encoded proteins, and this will probably represent the most challenging problem for the next generations of scientists.


**Comparative and Functional Genomics**
*Comp Funct Genom* 2003; **4**: 402–405.
Published online 18 July 2003 in Wiley InterScience (www.interscience.wiley.com). **DOI:** 10.1002/cfg.306

**Conference Review**

### Comparative modelling techniques: where are we?

Anna Tramontano*
*Department of Biochemical Sciences 'A. Rossi Fanelli', University of Rome 'La Sapienza', 00185 Rome, Italy*

**Correspondence to: Anna Tramontano, Department of Biochemical Sciences 'A. Rossi Fanelli', University of Rome 'La Sapienza', 00185 Rome, Italy. E-mail: anna.tramontano@uniroma1.it*

Received: 2 June 2003
Revised: 2 June 2003
Accepted: 3 June 2003

### Abstract

**The enormous increase in data availability brought about by genomic projects is paralleled by an equally unprecedented increase in the expectations for new medical, pharmacological, environmental and biotechnological discoveries. Whether or not we will be able to meet (at least partially) these expectations will depend on how well we will be able to interpret the data and translate the mono-dimensional information encrypted in genomes into a detailed understanding of its biological meaning at the phenotypic level. The process is far from being trivial, and the obstacles along the road are formidable: even the problem of identifying coding regions in eukaryotic genomes is not completely solved. Far more complex is identification of the function of the encoded proteins, and this will probably represent the most challenging problem for the next generations of scientists. Copyright © 2003 John Wiley & Sons, Ltd.**

**Keywords: comparative modelling; protein structure prediction; CASP; bioinformatics**

Biological function can be defined at several different levels, but in order to interfere with it for therapeutic or investigative purposes, we need to characterize it at the molecular level and to identify the precise role of specific amino acids and chemical groups. The problem is further complicated by the fact that function, rather than being the attribute of a single protein, is determined by the plethora of interactions that it establishes with other proteins and with the environment.

Although generally applicable methods for assigning function to a protein are not yet available, we are witnessing exciting advances in one of the fundamental steps of the process, the prediction of the three-dimensional structure of the native state of proteins.

The native structure of a protein represents the global free energy minimum that can be kinetically reached by the protein and, with rare exceptions, is solely determined by its amino acid sequence [1]. Our understanding of the energetic terms that govern the complex phenomenon of protein folding is not sufficiently complete to allow us to calculate the minimum free energy structure for a given amino acid sequence. The precision needed in the calculations should be sufficient to discriminate between the energy of the native state and that of any other conformation that the protein could assume, but proteins are only marginally stable, so that this difference only amounts to a few Kcal/mol, far beyond the precision that we can achieve today in our computations [2].

Leaving aside the idea of solving the problem on the basis of first principles, computational biology looked for, and found, other approaches based on the analysis of known protein structures, which represent a set of 'solved' examples to the protein folding problem.

The most important observation for protein structure prediction methods is that evolutionarily related proteins preserve their structure, to an extent dependent upon their evolutionary distance, and that evolutionary relationships are often detectable on the basis of the comparison of amino acid sequences. Therefore, if an evolutionary relationship between a protein of unknown structure and a protein of known structure can be detected from their sequences, then the latter will represent a

Copyright © 2003 John Wiley & Sons, Ltd.

suitable initial structural template for the former, and can be used to produce an atomic model of the unknown protein. Recent advances in this technique, known as comparative or homology modelling, will be discussed here. Although they are not covered here, interesting developments are also being observed in other heuristic methods, such as fold recognition, which try to recognize the fitness of a protein sequence for a protein architecture (independently of their evolutionary relationship), and in methods for predicting the structure of proteins with new folds (those that do not share any sequence or structural similarity with proteins of known structure).

Unbiased assessment of the quality of protein structure prediction methods has presented a problem for many years, because we need to test methods on proteins of unknown structure to ensure that the performance is not influenced by our previous knowledge of the protein, while, on the other hand, needing the experimental structure to assess the quality of a model. The prediction community has therefore established an experiment (Critical Assessment of Methods for Protein Structure Prediction; CASP) aimed at assessing prediction methods in an unbiased and comprehensive way [3–5].

The CASP experiment has been run every 2 years since 1994 and is a multi-step process that lasts a few months. First, structural biologists are asked to release the amino acid sequence of proteins, the CASP targets, whose structures are likely to be completed before the meeting. Next, scientists predict the structure of the target proteins and deposit their predictions before the experimental structures are released. Finally, the targets, the models and a numerical evaluation of the quality of the predictions are made available to independent assessors who are asked to critically evaluate the results, draw conclusions about the state of the art in the field of protein structure prediction and report on their analysis at the CASP meeting in December.

In the last CASP experiment, I assessed the models produced by comparative modelling methods with the invaluable assistance of Veronica Morea [7]. The details of the assessment are published elsewhere [7] and the data are available via the CASP Web site (**http://www.predictioncenter.llnl.gov**).

The large number of groups participating (265) and the huge number of deposited models (28 728) testify to the interest of the scientific community in the CASP experiment, but also make the assessment procedure quite heavy and, especially, do not allow every model to be visually inspected. Therefore, models are automatically compared with their target structures and the parameters derived from these comparisons are statistically evaluated, so that visual inspection can be limited to those models that are deemed to be particularly interesting [6,7].

CASP results are relevant both for the biological community at large, who can use the results to estimate the reliability of structure prediction methods, and for predictors, who can benchmark their methods and identify the areas where future efforts can be more productively focused.

As far as the first aspect is concerned, two interesting and useful conclusions could be derived from the latest experiment.

As mentioned before, the expected quality of a homology model depends upon the evolutionary distance between the target and the template, which can be estimated by the percentage sequence identity between the two protein sequences. On this basis, we can roughly divide targets into three categories: easy (sequence identity above 40%), hard (sequence identity below 30%) and intermediate.

Figure 1 shows the difference between the quality of the best and average models submitted to CASP5 for each target. It is clear that for easy and intermediate targets, the average quality of a model is not very different from the best one. In other words, the majority of the groups are able to produce models of satisfactory quality. This is an important point, in my view, since it demonstrates that, in most cases of interest for molecular biologists, the available methods are sufficiently accurate to be used as guides for designing and interpreting experiments.

The CASP targets are also submitted to automatic servers freely available on the network in order to verify the state of the art of tools that can be used by experimentalists without a specific expertise in modelling techniques.

Figure 2 shows the average score obtained by the best 50 groups, and highlights those corresponding to automatic servers, which account for 10% of them. This indicates that some servers, listed in

Copyright © 2003 John Wiley & Sons, Ltd. 

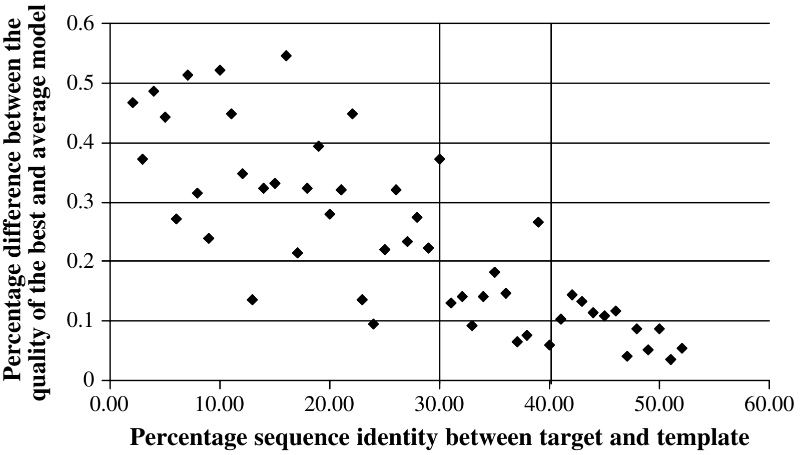

**Figure 1.** The difference between the best and average models for each target, as a function of the percentage of sequence identity with its closest structural template (this gives an estimate of the difficulty of the targets). The quality of models is calculated using the GDT-TS parameter defined as 1/4 (GDT-1 + GDT-2 + GDT-4 + GDT-8), where GDT-*n* is the number of residues of the model within *n* Å from the corresponding residue in the target structure

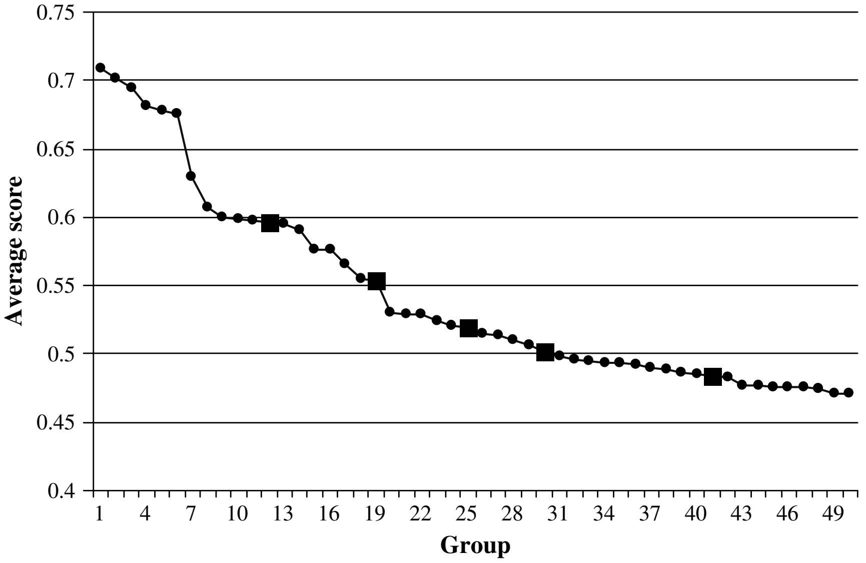

**Figure 2.** The average scores achieved by the best 50 groups participating in CASP5. The five best non-server groups who submitted more than 10 models are Murzin, VENCLOVAS, Bujnicki-Janusz, Ginalski and GeneSilico (indicated by the names they used to register to the experiment). The results of these five groups are statistically indistinguishable. Black squares correspond to the servers 3D-SHOTGUN-3DS5, 3D-SHOTGUN-3DS3, BAKER-ROBETTA, Pmodel and 3D-SHOTGUN-INBGU (listed in decreasing order of their score). The score for a model is defined as its Z-score with respect to the distribution of the GDT-TS values obtained by all groups for the same target. The reported score for each group is the average score for all submitted models the legend to the figure, have a quite high reliability. Unfortunately, there are servers that perform rather poorly (data not shown). Experimentalists are strongly advised to consult the summary of the results of the participating servers at the CASP website and to consider these data when selecting a structure prediction server.

We believe that the results summarized here and described in more detail in the assessment report for comparative modelling in CASP [7], represent

Copyright © 2003 John Wiley & Sons, Ltd.

very good news for the scientific community at large. They demonstrate that structure prediction methods are sufficiently mature and robust to become part of the suite of useful tools that computational biology has made available to the biological community, such as database search facilities.

The results of CASP5 have also highlighted another important aspect that has greatly attracted the attention of the prediction community.

In general, a homology modelling procedure requires several steps: the selection of the evolutionarily related protein of known structure; the sequence alignment of the two protein sequences to try to infer the correct structural correspondence between their amino acids; the prediction of the conformation of regions that are structurally divergent between the target and the template (e.g. where insertions and deletions of amino acids are observed); the prediction of the conformation of the side chains; and the final optimization of the model. One major problem that has to be faced is that the quality of a model, although dependent on each of the described steps, can only be estimated at the end of the whole procedure. For example, it is possible that one of the insertions cannot be modelled convincingly, given the rest of the model, or that some side chains will be incompatible with each other in the selected alignment. The only possibility at this stage is to trace back the cause of the difficulty and start all over again, e.g. using a different template or modifying the alignment, and this is clearly a very unsatisfactory solution.

Thanks to the availability of reliable automatic methods for protein structure prediction, also fostered by previous CASP results, it is now possible to construct, with limited effort, several initial models, e.g. using different templates and/or slightly different alignments with each template and/or different methods for modelling structurally divergent regions. At the end of the procedure, the quality of the different models can be evaluated at the atomic level, not only avoiding having to repeat the procedure in the case of problems but, more importantly, permitting the retention of any of the many alternative choices that are possible for each of the steps of the procedure.

Perhaps not surprisingly, the predictors who used such an approach in CASP5 were the most successful ones. Notably, there are already automatic servers (the so-called meta-predictors) that use a similar procedure in an automatic, user-transparent fashion.

The choice of the final model clearly depends upon the method used for assessing the quality of the several alternative ones produced and it is quite easy to predict that in the near future the efforts of the community will be focused upon this aspect of the problem. Interestingly, this problem is also relevant for other methods for protein structure prediction, such as fold recognition methods or methods for predicting new folds. We are already witnessing a progressive merging of the different prediction communities and it can easily be foreseen that they will effectively cross-fertilize each other.

### Acknowledgements

I would like to thank Veronica Morea for her help in the assessment of the CASP models, Adam Zemla for providing the numerical evaluation of the models, the CASP organizers, and all of the predictors and structural biologists who made CASP possible.

### References

1. Anfinsen CB, Harrington WF, Hvidt A, Lindstrom-Lang K. 1955. Studies on the structural basis of ribonuclease activity. *Biochim Biophys Acta* **17**: 141–142.
2. Finkelstein A. 1997. Protein structure: what is it possible to predict now? *Curr Opin Struct Biol* **7**: 60–71.
3. Moult J, Fidelis K, Zemla A, Hubbard T. 2001. Critical assessment of methods of protein structure prediction (CASP): round IV. *Proteins* **45**(suppl): 2–7.
4. Moult J, Hubbard T, Bryant SH, Fidelis K, Pedersen JT. 1997. Critical assessment of methods of protein structure prediction (CASP): round II. *Proteins* **29**(suppl): 2–6.
5. Moult J, Hubbard T, Fidelis K, Pedersen JT. 1999. Critical assessment of methods of protein structure prediction (CASP): round III. *Proteins* **37**(suppl): 2–6.
6. Tramontano A, Leplae R, Morea V. 2001. Analysis and assessment of comparative modelling predictions in CASP4. *Proteins* **45**(suppl 5): 22–38.
7. Tramontano A, Morea V. 2003. Analysis and assessment of comparative modelling predictions in CASP5. *Proteins* **52**(suppl 7): (in press).

Copyright © 2003 John Wiley & Sons, Ltd. 
